# Factors Affecting Breastfeeding Practices in Sindh Province, Pakistan: A Secondary Analysis of Cross-Sectional Survey Data

**DOI:** 10.3390/ijerph16101689

**Published:** 2019-05-14

**Authors:** Jin-Won Noh, Young-mi Kim, Nabeel Akram, Ki-Bong Yoo, Jooyoung Cheon, Lena J. Lee, Young Dae Kwon, Jelle Stekelenburg

**Affiliations:** 1 Department of Healthcare Management, Eulji University, Seongnam 13135, Korea; jw.noh@eulji.ac.kr; 2 Global Health Unit, Department of Health Sciences, University Medical Centre Groningen, University of Groningen, 9713 GZ Groningen, The Netherlands; jelle.stekelenburg@online.nl; 3 Jhpiego, Johns Hopkins University, Baltimore, MD 21231, USA; Young-Mi.Kim@jhpiego.org (Y.-m.K.); nabeel.akram@jhpiego.org (N.A.); 4 Department of Health Administration, Department of Information & Statistics, Yonsei University, Wonju 26493, Korea; ykbong@yonsei.ac.kr; 5 Department of Nursing Science, Sungshin University, Seoul 01133, Korea; jcheon@sungshin.ac.kr; 6 National Institutes of Health Clinical Center, Bethesda, MD 20892, USA; jumin.park@nih.gov; 7 Department of Humanities and Social Medicine, College of Medicine and Catholic Institute for Healthcare Management, The Catholic University of Korea, Seoul 06591, Korea; 8 Department of Obstetrics and Gynecology, Medical Centre Leeuwarden, 8934 AD Leeuwarden, The Netherlands

**Keywords:** breastfeeding, Pakistan, maternal and child health, socioeconomic status

## Abstract

Breastfeeding practices are critical for child health and growth. This paper investigates demographic factors, socioeconomic status, and information sources that affect breastfeeding practices in Sindh Province, Pakistan. A secondary analysis was performed of data on 10,028 women with a birth in the preceding two years who had participated in the 2013–14 Maternal and Child Health Program Indicator Survey. Multiple logistic regressions were used to test the association between breastfeeding status (ever breastfed and still breastfeeding) and age, number of living children, residence, education, wealth, information sources about breastfeeding, assistance during delivery, and place of delivery. Of the 9955 women included in the analysis, 97.9% had breastfed and 83.9% were still breastfeeding at the time of the survey. Being in the second, third, or fourth wealth quintiles and receiving breastfeeding information from relatives and friends were associated with ever breastfeeding. Women who were 35 years or older, living in a town/small city, higher maternal education, middle wealth quintile, and receiving breastfeeding information from the media were associated with still breastfeeding. The findings suggest the need to develop interventions considering maternal socioeconomic status and peer counseling interventions. Mass media campaigns to promote breastfeeding practices should be accompanied by governmental restrictions on the marketing of infant formula.

## 1. Introduction

Optimal breastfeeding practices improve the survival, health, and development of children [1]. Benefits of breast milk include enhanced cognitive development [2], an optimized immune system [3], reduced risk of some autoimmune and atopic diseases [4,5], obesity [6] and leukemia [7] of children. In 2016, a *Lancet* series on breastfeeding estimated that 823,000 deaths of children under the age of five years could be prevented every year through optimal breastfeeding practices [8]. However, according to the *Global Breastfeeding Scorecard*, which evaluated 194 nations, the current global rate of exclusive breastfeeding is still unsatisfactory (40 percent of infants under six months of age) and only 23 of the countries have exclusive breastfeeding rates above 60 percent although overall rates of continued breastfeeding at one year are higher (74%) [9]. 

Given the benefits of breastfeeding infants, it is important to identify which factors may influence breastfeeding practices and help to develop effective intervention programs [10]. A growing body of literature reports that, maternal socioeconomic factors are related to breastfeeding practices in both low and middle income countries [11,12,13,14,15,16,17,18]. Maternal education has been reported to be a significant predictor of breastfeeding practices; mothers with a low level of education are at high risk of stopping exclusive breastfeeding [11,13,17,19,20]. Several studies also found a negative association between household income and the practice of exclusive breastfeeding [15,19,20]. Institutional delivery and antenatal care have been found to predict exclusive breastfeeding [11,20], while advertisements and articles promoting breastfeeding are positively correlated with increasing breastfeeding rates [18]. 

Pakistan has a frighteningly low rate of exclusive breastfeeding, with only 38 percent of infants less than six months being exclusively breastfed [21]. However, we have limited knowledge on how to influence the breastfeeding practices of Pakistani women. In particular, no published studies have assessed important modifiable factors that may be associated with breastfeeding practices in Pakistan, such as information about breastfeeding or healthcare during pregnancy and delivery. Therefore, a population-based study of Pakistani women may be helpful to understand breastfeeding practices and the factors that may influence their desire to breastfeed. Healthcare providers and policy makers could use such information to design and develop successful interventions, such as peer counseling, lactation consultation, and breastfeeding education. 

The purpose of this analysis was to measure breastfeeding practices in Sindh province, Pakistan and to identify determinants, demographic factors, socioeconomic status, and information sources that affect breastfeeding practices.

## 2. Methods

### 2.1. Data and Subjects

This paper presents a secondary analysis of data collected by the Maternal and Child Health Program Indicator Survey from recent mothers in Sindh province, Pakistan in 2013 and 2014 [22]. The survey was cross-sectional and used a multi-stage, stratified sampling design. Data were collected for two years. The sampling frame was developed by urban and rural categorization based on the national census of Pakistan. First, disproportionate sampling was used to allocate samples in different districts; then the probability proportional to size method was used to select the required number of cities or villages in each district [22]. After selection of the cities or villages, interviews were conducted with a maximum of 10 random subjects in each city in urban areas or each village in rural areas. Data were collected on respondents’ socioeconomic status, reproductive history, health knowledge, pregnancy experience, breastfeeding practices, child health, and fertility preferences, along with mass media and communication exposure. 

The total survey population consisted of 10,200 women who had a live birth within the previous two years; their answers to the survey questions pertained to their last live birth. This analysis excludes women with missing information on number of living children (*n* = 23), wealth (*n* = 34), education level (*n* = 22), husband’s education level (*n* = 93), baby’s age (*n* = 73), reducing the sample size to 9955 women.

The Maternal and Child Health Program Indicator Survey was approved by the Johns Hopkins University School of Public Health Institutional Review Board (IRB00005002) and the National Bioethics Committee of Pakistan. The analysis reported here used secondary data; there was no direct intervention and it was not possible to identify individual respondents. 

### 2.2. Variables

The dependent variables in this analysis were *ever breastfed* and *still breastfeeding*. Ever breastfed was defined by the mother’s yes or no response to the question, “Did you ever breastfeed?” and captured whether respondents had any breastfeeding experience or not about respondent’s last pregnancy that resulted in a live birth within past two years. Still breastfeeding was defined by the mother’s yes or no response to the question, “Are you still breastfeeding?” This question was only asked to respondents whose child was still living at the time of the survey. Among the 9955 women in the sample, 105 reported their child had died; an additional 224 women had missing answers for questions about still breastfeeding and/or whether the child was still living. Therefore, the analysis for still breastfeeding is limited to 9626 women.

Independent variables were selected based on a systematic review of factors associated with breastfeeding in developing countries [23]. They included baby’s age, maternal age, number of living children, socioeconomic status, source of information about breastfeeding, healthcare during pregnancy and delivery and survey year. Baby’s age at the time of the survey was included as a variable and based on the World Health Organization breastfeeding guideline, categorized into 0–6 months, 7–12 months, 13–18 months and more than 18 months.

We examined residence (rural, town/small city, large city), woman’s education, husband’s education, and wealth quintiles as socioeconomic status. Wealth quintiles were calculated from household assets using principal components analysis [24]. 

The survey asked women, “During the last 12 months have you received any information about breastfeeding from the following sources?” Sources were categorized as health professionals (doctors, nurse/midwives and female health visitors), low-level health workers (Dai-traditional birth attendants, female health workers, homeopaths, Hakim-herbal medicine practitioners and outreach workers), media (television, radio, telephone helpline, text message, health education/awareness session and print media) and relatives/friends. Respondents were able to choose multiple categories. 

To assess healthcare during pregnancy and delivery, we included the person who attended the delivery and the place of delivery as variables. Attendance during delivery was classified into traditional birth attendant, health professional and no one/others. Place of delivery was classified into home, private facility, and public facility.

### 2.3. Statistical Analysis

We used chi-squared tests to conduct bivariate analyses for general distribution between ever breastfed or still breastfeeding and independent variables. Then we conducted multiple binary logistic regressions for each of the dependent variables: ever breastfed and still breastfeeding. Odds ratios (ORs) and 95% confidence intervals (CIs) were estimated for multiple binary logistic regressions to measure the association between independent variables and each dependent variable. The significance level was set at 0.05. Survey weights were applied to all analyses. All analyses were performed using SAS version 9.4 (SAS Institute, Inc., Cary, NC, USA).

## 3. Results

At the time of the survey, 97.9% of women had ever breastfed and 83.9% of women were still breastfeeding. Residence and wealth quintile were associated with both dependent variables. Baby’s age, education level, wealth, health professional, media, attendance during delivery, and survey year were associated with still breastfeeding. The still breastfeeding rate was higher when women lived in a large city (85.3%), had received no education (85.8%), were in the lowest wealth quintile (87.1%), received no information from health professional (84.6%) or media (85.1%), received attendance during delivery from a traditional birth attendant (85.7%) and were surveyed in 2013 (86.2%) (Table 1).

Table 2 shows the factors associated with ever breastfed and still breastfeeding using multiple logistic regressions. The references of both dependent variables were ‘no’; therefore, an OR over one suggests that a factor is associated with ever breastfeeding or still breastfeeding. The odds that women ever breastfed were significantly lower for women in the second, third (middle), fourth wealth quintiles; they were significantly higher for women who received breastfeeding information from friends or relatives (OR = 1.72, 95% CI = 1.24–2.39). The odds that women were still breastfeeding at the time of the survey were significantly greater for women who were 35 years or older (OR = 1.36, 95% CI = 1.11–1.66) and living in towns or small cities (OR = 1.22, 95% CI=1.01–1.49); they were significantly lower for women who had more education, were in the middle wealth quintile (OR = 0.76, 95% CI = 0.610.95), had received breastfeeding information from the media (OR = 0.82, 95% CI = 0.71–0.95) (Table 2).

## 4. Discussion

The 2013-14 Maternal and Child Health Program Indicator Survey found very high levels of breastfeeding in Sindh province. Virtually all women (97.9 %) had some experiences of breastfeeding, but the odds of women still breastfeeding decreased rapidly during the next six months from baby’s birth, while World Health Organization (WHO) recommended mothers to breastfeed up to the age of two years or beyond [1]. The high percentage of mothers who were still breastfeeding in this study (83.9%) did not indicate that babies older than six months meet their nutritional requirements. Unfortunately, we could not examine or adjust for the type and quality of breastfeeding practices including early initiation of breastfeeding, exclusive breastfeeding, prelacteal and supplemental feeding. These are essential WHO recommendations, which advise that breastfeeding should begin within the first hour of life and exclusive breastfeeding should be sustained for six months after birth [1]. In previous studies of Pakistan, the percentages of women to sustain exclusive breastfeeding for six months ranged from 38% to 54% [24,25] and the median duration of exclusive breastfeeding was less than one month [24]. These findings suggest that breastfeeding mothers may not engage in best practices, as babies grow older. Therefore, at least once every six months, post-natal follow-up support for all breastfeeding women are needed to find women who consider discontinuing breastfeeding and encourage them to breastfeed for at least two years with appropriate complementary food based on the WHO recommendations. 

In this study, women who live in town/small city showed higher odds of breastfeeding their babies for longer periods than those who live in rural, while living in large city was not a determinant of still breastfeeding. A study reported that Pakistani mothers with babies under six months who live in suburban areas showed 61% of exclusive breastfeeding, which was higher than 47% of mothers who live in urban areas, which may be due to non-affordability of the prepared formula milk and cultural values [25]. Most rates for health care utilization and public health practice among women living in town/small city were between those living in large city and rural areas in Pakistan [22], which indicated that women living in town/small city were influenced by both urban and rural environments. Factors associated with the use of medical facility as the place of birth in town/small city were different from those in large city or rural areas [26]. Therefore, further studies should determine unique characteristics of women in town/small city and it is necessary to approach them with different maternal and child health promotion strategies based on the results of those studies. 

Findings from previous studies about the relationship between maternal education and breastfeeding practices are inconsistent [12,13,14,17,27,28]. In this study, maternal education was not a significant determinant of starting breastfeeding, which was aligned with the findings of a previous study conducted in two rural districts of Sindh, Pakistan [29], but it was negatively associated with longer breastfeeding. The inconsistency in findings about the relationship between maternal education and breastfeeding practices may be due to cultural beliefs regarding prelacteal feeding, that is, the practice of giving food to newborns before the initiation of breastfeeding [25,30,31] and the misconception that breastfeeding is a cause of weakness in mothers [25,31]. Educated women were more likely to deliver in a health facility [24], but 74.6% of women who deliver in a health facility provided prelacteal feed, which was similar to 75.8% of women who deliver at home [24]. Another possible reason for the negative association between maternal education and breastfeeding may be that women with higher education have more opportunities to engage in formal employment and control over their cash earnings, which leads to less still breastfeeding due to their affordability [16,17,24,28,32]. According to the Pakistan Demographic and Health Survey 2012–13 [24], the majority of highly educated women were employed at the professional/technical/managerial job, whereas the majority of uneducated women were employed at the unskilled manual or employed in agriculture. Possibly, more highly educated women have jobs away from home, which makes it unpractical to breastfeed because they are away for long hours.

Interestingly, women in the second, third (middle), fourth wealth quintiles were less likely to have ever breastfed than those in the poorest wealth quintile, but being in the richest wealth quintile was not a significant determinant of breastfeeding practices. Previous studies have noted that infant formula and cow milk are not affordable for poor mothers so they are more likely to engage in exclusive breastfeeding [15,29]. Mothers and fathers who participated in focus group discussions in Punjab province, Pakistan, believed that wealthy people bought expensive milk to show their wealth [31]. In contrast, a study from Nigeria reported that more mothers from wealthier households practiced exclusive breastfeeding than those from poorer households, which may reflect better health care access for wealthier mothers [13]. The findings about the relationship between five wealth quintiles and breastfeeding practices in this study may be due to a difference in the distribution of occupations among the wealth quintiles. The two common occupations in the fifth (richest) quintile were the professional/technical/managerial job and sales/services, whereas three common occupations in the first to fourth wealth quintiles were sales/services, unskilled manual and agriculture [24]. Program managers may need to develop appropriate interventions for women after considering women’s economic status and occupational characteristics simultaneously in Pakistan. 

This analysis revealed a negative association between receiving information from the media and still breastfeeding. A previous intervention study in Sindh examined the effect of mass media campaigns (mainly television spots) on breastfeeding practices. It found that the proportion of mothers who received information about the importance of early breastfeeding initiation and exclusive breastfeeding increased significantly, but there was no significant improvement in breastfeeding practices. This suggested that mass media campaigns may have been helpful in delivering information and driving conversations about breastfeeding to Pakistani women but did not lead to changes in actual practice [33]. The adverse effect of mass media on breastfeeding practices documented here may be explained by research on the impact of marketing breast milk substitutes on breastfeeding practices in low- and middle-income countries [34]. Companies advertise infant formula as a breast milk substitute that is modern and better than breast milk via television, radio, print advertisements, internet websites and social media [35]. The prevalence of mothers who recalled advertisement or promotion of breast milk substitutes on television was 28% in 2012 in Pakistan [35]. Even though Pakistan adopted the International Code of Marketing of Breast-milk Substitutes in 2002 to restrict the marketing of infant formula [36,37], most healthcare providers were not aware of the law and the marketing of formula may have continued after the law was applied [37]. Our findings suggest that mass media campaigns to improve breastfeeding practices in Pakistan will not have a positive impact unless they are accompanied by the government restrictions on the marketing of infant formula. 

We found that receiving breastfeeding information from relatives or friends had a positive impact on starting breastfeeding. This may be due to the nature of their advice. In the Muslim community, relatives or friends are the main people providing breastfeeding advice [30,38]. An intervention study conducted in urban Bangladesh has highlighted the need for community-based peer counseling using trained community female volunteers to promote good breastfeeding practices. When peer counseling at home included other family members, mothers were five times more likely to practice exclusive breastfeeding for up to five months compared to mothers who received no intervention [38]. Peer counseling also had a positive effect on early initiation of breastfeeding within the first hour of birth [38]. Peer counseling interventions may be tested in Pakistan to improve early breastfeeding initiation and exclusive breastfeeding. 

This analysis has several limitations. First, due to the cross-sectional nature of the survey, causal relationships with determinants cannot be ascertained. Second, questions about breastfeeding were dichotomized to yes/no answers, so there is no information on when breastfeeding was initiated after birth, the duration of exclusive and non-exclusive breastfeeding and the practice of prelacteal feeding, all of which were significant elements in previous studies of breastfeeding behaviors [1,33,38]. Third, the findings cannot be generalized to all mothers in Pakistan because the survey was limited to a single province, Sindh. 

However, the finding about negative association between baby’s age and still breastfeeding rate suggests the need to follow-up breastfeeding mothers to continue breastfeeding at least once every six months. This analysis also found an association between the socioeconomic status of women and good breastfeeding practices and suggests the need to develop better-shaped interventions in line with maternal educational level and economic status. In countries where Islam is the dominant religion, relatives or friends could be a good support to promote breastfeeding. Therefore, public health officials and health care providers should consider peer counseling interventions targeting relatives and friends.

## 5. Conclusions

This analysis provides evidence for further efforts to improve breastfeeding practices in Sindh province, Pakistan by identifying factors associated with breastfeeding. Policy makers and program managers need to develop targeted interventions to improve breastfeeding practices based on the baby’s age and maternal socioeconomic status. This analysis highlights the role of mass media and relatives/friends as information sources about breastfeeding. Mass media campaigns to improve breastfeeding practices should be accompanied by the governmental restriction of marketing of formula milk in Pakistan. In addition, it is necessary to consider development of peer counseling interventions to improve breastfeeding practices. 

## Figures and Tables

**Table 1:** Characteristics of survey respondents by breastfeeding status (n = 9955).

Characteristic		Ever Breastfed			Still Breastfeeding		
		Number	Yes (Weighted %)	*p*-Value	Number	Yes (Weighted %)	*p*-Value
*Demographics*							
Baby’s age, in months	0–6	2251	98.4	0.260	2173	95.0	<0.001
	7–12	3056	97.7		2956	90.0	
	13–18	2677	98.1		2586	81.7	
	19–	1971	97.7		1911	64.4	
Woman’s age, in years	15–24	2994	97.8	0.524	2896	83.8	0.148
	25–34	5586	98.0		5405	83.5	
	35–	1375	98.3		1325	85.8	
Number of living children	1	6017	97.8	0.241	5811	84.3	0.424
	2	2249	98.0		2174	83.4	
	3–	1689	98.4		1641	83.2	
*Socioeconomic*							
Residence	Rural	2552	97.9	0.006	2472	81.7	<0.001
	Town/small city	2659	97.0		2551	85.1	
	Large city	4744	98.3		4603	85.3	
Woman’s education	No education	5927	98.1	0.568	5725	85.8	<0.001
	Primary or middle	2043	98.0		1982	81.3	
	Secondary or higher	1985	97.7		1919	81.7	
Husband’s education	No education	3801	97.8	0.752	3677	84.9	0.090
	Primary or middle	2224	98.2		2143	83.8	
	Secondary or higher	3930	97.9		3806	83.0	
Wealth quintile	First (poorest)	2004	99.0	0.007	1957	87.1	<0.001
	Second	1987	97.4		1915	85.8	
	Third (middle)	1978	97.9		1904	82.6	
	Fourth	1985	97.6		1913	82.6	
	Fifth (richest)	2001	98.0		1937	82.1	
*Information sources about breastfeeding*							
Health professional	No	6391	97.8	0.218	6160	84.6	0.019
	Yes	3564	98.2		3466	82.8	
Low-level health workers †	No	7180	97.9	0.838	6935	83.9	0.846
	Yes	2775	98.0		2691	83.8	
Media	No	7819	98.0	0.473	7552	85.1	<0.001
	Yes	2136	97.8		2074	79.9	
Relatives/friends	No	5522	97.5	<0.001	5309	84.1	0.531
	Yes	4433	98.5		4317	83.7	
*Healthcare during pregnancy and delivery*							
Assistance during delivery	Traditional birth attendant	3052	98.1	0.479	2953	85.7	0.012
	Health professional	6769	97.9		6542	83.2	
	No one/others	134	99.3		131	83.6	
Place of delivery	Home	3448	98.1	0.455	3333	85.1	0.096
	Private facility	4901	97.8		4736	83.3	
	Public facility	1606	98.2		1557	83.6	
*Survey year*	2013	3897	97.6	0.052	3734	86.2	<0.001
	2014	6058	98.2		5892	82.4	

^†^ Low-level health workers include Dai-traditional birth attendants, lady health workers, homeopaths, Hakim-herbal medicine practitioners and outreach workers.

**Table 2:** Factors associated with breastfeeding status in multiple logistic regressions.

Factors		Ever Breastfed (ref = no)		Still Breastfeeding (ref = no)	
		OR	95% CI	OR	95% CI
*Demographic*					
Baby’s age, in months	0–6	1.00		1.00	
	7–12	0.70	(0.47–1.05)	0.48	(0.39–0.61)
	13–18	0.83	(0.54–1.27)	0.23	(0.19–0.29)
	19–	0.65	(0.42–1.01)	0.09	(0.07–0.12)
Woman’s age, in years	15–24	1.00		1.00	
	25–34	1.11	(0.82–1.51)	1.12	(0.98–1.27)
	35–	1.43	(0.87–2.35)	1.36	(1.11–1.66)
Number of living children	1	1.00		1.00	
	2	1.21	(0.85–1.71)	1.08	(0.93–1.24)
	3–	1.48	(0.98–2.25)	1.03	(0.88–1.20)
*Socioeconomic*					
Residence	Rural	1.00		1.00	
	Town/small city	0.77	(0.51–1.18)	1.22	(1.01–1.49)
	Large city	1.22	(0.76–1.95)	1.18	(0.97–1.45)
Woman’s education	No education	1.00		1.00	
	Primary or middle	1.00	(0.67–1.48)	0.77	(0.66–0.91)
	Secondary or higher	0.81	(0.52–1.28)	0.77	(0.63–0.93)
Husband’s education	No education	1.00		1.00	
	Primary or middle	1.36	(0.92–2.00)	1.05	(0.90–1.23)
	Secondary or higher	1.32	(0.91–1.91)	1.11	(0.95–1.30)
Wealth quintile	First (poorest)	1.00		1.00	
	Second	0.40	(0.23–0.69)	0.92	(0.75–1.12)
	Third (middle)	0.53	(0.29–0.98)	0.76	(0.61–0.95)
	Fourth	0.49	(0.25–0.97)	0.86	(0.66–1.11)
	Fifth (richest)	0.57	(0.27–1.22)	0.89	(0.66–1.19)
*Information sources about breastfeeding*					
Health professional	No	1.00		1.00	
	Yes	1.07	(0.77–1.50)	1.00	(0.88–1.14)
Low-level health workers†	No	1.00		1.00	
	Yes	0.85	(0.60–1.21)	0.98	(0.85–1.13)
Media	No	1.00		1.00	
	Yes	0.83	(0.58–1.19)	0.82	(0.71–0.95)
Relatives/friends	No	1.00		1.00	
	Yes	1.72	(1.24–2.39)	1.13	(0.99–1.29)
*Healthcare during pregnancy and delivery*					
Assistance during delivery	Traditional birth attendant	1.00		1.00	
	Health professional	0.92	(0.46–1.84)	0.78	(0.57–1.01)
	No one/others	3.11	(0.35–27.99)	0.82	(0.48–1.39)
Place of delivery	Home	1.00		1.00	
	Private facility	0.99	(0.51–1.95)	1.20	(0.91–1.59)
	Public facility	1.27	(0.61–2.66)	1.26	(0.93–1.69)
*Survey year*	2013	1.00		1.00	
	2014	1.20	(0.88–1.63)	0.91	(0.80–1.04)

Ref, reference; OR, odds ratio; CI, confidence interval, ^†^ Low-level health workers included Dai-traditional birth attendants, lady health workers, homeopaths, Hakim-herbal medicine practitioners, and outreach workers.
